# Connexons Coupling to Gap Junction Channel: Potential Role for Extracellular Protein Stabilization Centers

**DOI:** 10.3390/biom12010049

**Published:** 2021-12-30

**Authors:** László Héja, Ágnes Simon, Zsolt Szabó, Julianna Kardos

**Affiliations:** Research Centre for Natural Sciences, Institute of Organic Chemistry, Magyar Tudósok Körútja 2, 1117 Budapest, Hungary; agnes.simon@ttk.hu (Á.S.); szabo.zsolt@ttk.hu (Z.S.); kardos.julianna@ttk.hu (J.K.)

**Keywords:** Cx43 GJC with two membranes, GJ architecture, close/open disulfide bonds preconditions, fluctuation dynamics of protein stabilization centers (SCs), cystine disulfide related GJ SC patterns, subtype-specific SC motifs

## Abstract

Connexin (Cx) proteins establish intercellular gap junction channels (Cx GJCs) through coupling of two apposed hexameric Cx hemichannels (Cx HCs, connexons). Pre- and post-GJ interfaces consist of extracellular EL1 and EL2 loops, each with three conserved cysteines. Previously, we reported that known peptide inhibitors, mimicking a variety of Cx43 sequences, appear non-selective when binding to homomeric Cx43 vs. Cx36 GJC homology model subtypes. In pursuit of finding potentially Cx subtype-specific inhibitors of connexon-connexon coupling, we aimed at to understand better how the GJ interface is formed. Here we report on the discovery of Cx GJC subtype-specific protein stabilization centers (SCs) featuring GJ interface architecture. First, the Cx43 GJC homology model, embedded in two opposed membrane bilayers, has been devised. Next, we endorsed the fluctuation dynamics of SCs of the interface domain of Cx43 GJC by applying standard molecular dynamics under open and closed cystine disulfide bond (**^C^**S-S**^C^**) preconditions. The simulations confirmed the major role of the unique trans-GJ SC pattern comprising conserved (55**N**, 56**T**) and non-conserved (57Q) residues of the apposed EL1 loops in the stabilization of the GJC complex. Importantly, clusters of SC patterns residing close to the GJ interface domain appear to orient the interface formation via the numerous SCs between EL1 and EL2. These include central ^54**C**^S-S^198**C**^ or ^61**C**^S-S^192**C**^ contacts with residues 53R, 54**C**, 55**N**, 197**D**, 199**F** or 64**V**, 191**P**, respectively. In addition, we revealed that GJC interface formation is favoured when the psi dihedral angle of the nearby 193**P** residue is stable around 180° and the interface SCs disappear when this angle moves to the 0° to −45° range. The potential of the association of non-conserved residues with SC motifs in connexon-connexon coupling makes the development of Cx subtype-specific inhibitors viable.

## 1. Introduction

Vertebrate cells perform dominant remodelling of intercellular adhesion and communication via specific subunit combinations of hexameric connexin (Cx) hemichannels (Cx HCs, connexons) that couple to form dodecameric gap junction channels (Cx GJCs) [1,2,3,4,5,6,7,8].

Canonical Cx proteins present three conserved cysteines in their extracellular loops EL1 and EL2, except Cx23 subtype identified in mammals and zebrafish. The Cx23a isoform has only two conserved cysteines in its extracellular EL1 and EL2 loops and is characterized by less efficient GJC coupling [9]. Pannexin [10] and innexin [11] channels, also releasing small molecules and ions contain two conserved cysteines in EL1 and EL2 loops and they hardly serve GJC functions either. Innexin 4 (CHEM-7) [12] is an exception, as it contains three cysteine residues per subunits enabled to form synaptic GJCs, specifically affecting chemotaxis behaviour [13]. Therefore, we may conjecture the implementation of the GJC function with the higher number of extracellular cysteines in EL1 and EL2 loops. An exception to this rule is the Cx31.3 isoform that does not form functional GJC [5] despite the presence of three cysteines in each connexin. This exceptional property may be attributed to the mutations 55N→55H and 197D→197T at the HC/HC interface (*cf.* Results section). Based on mapping mutation-function relationships, Foote and co-workers [14] suggested that all **^C^**S-S**^C^** bonds take part in anti-parallel beta sheets with the first **C**(1) cysteine in one loop connected to the **C**(3) of the other. Distances of cysteines **C**(1), **C**(2) and **C**(3) in EL1 and EL2 loop motifs may also anticipate a critical role for cystine disulfide **^C^**^(2)^S-S**^C^**^(2)^ bond formation.

Previously, we reported that known peptide inhibitors, mimicking a variety EL1 or EL2 sequences of Cx43 subtype, appear non-selective when binding to Cx43 versus Cx36 GJC subtypes [15]. In searching for potentially Cx subtype-specific inhibitors, we devised a strategy to design protein components and/or small molecules involved in the inhibition of protein-protein interactions [16,17,18]. Here we explore the prospective approach to inhibit connexon coupling by targeting distinguishable Cx subype-specific structural motifs within the GJ domain.

To this end, we seek unique EL1 and/or EL2 patterns of Cx GJC by selecting the homomeric Cx43 GJC as prototype. Cx43 GJCs are widespread in the brain, connecting the brain-specific astrocytic cells [19,20]. Also, Cx43 GJCs are bound with a variety of tissue cell contacts outside the brain, including heart [21] and kidney [22]. Moreover, Duchenne muscular dystrophy was accompanied by aberrantly enhanced Cx43 in both cardiac and skeletal muscles [23]. In addition to neocorticogenesis [24], astrocytic Cx43 GJCs are responsible for long-range synchronized neural activity underlying epilepsy [25,26] and memory-associated slow-wave sleep [27], whereas neuronal Cx36 GJCs distinguish fast synchronization by forming electrical synapses (SI Table S1).

## 2. Results

**Figure 1:**
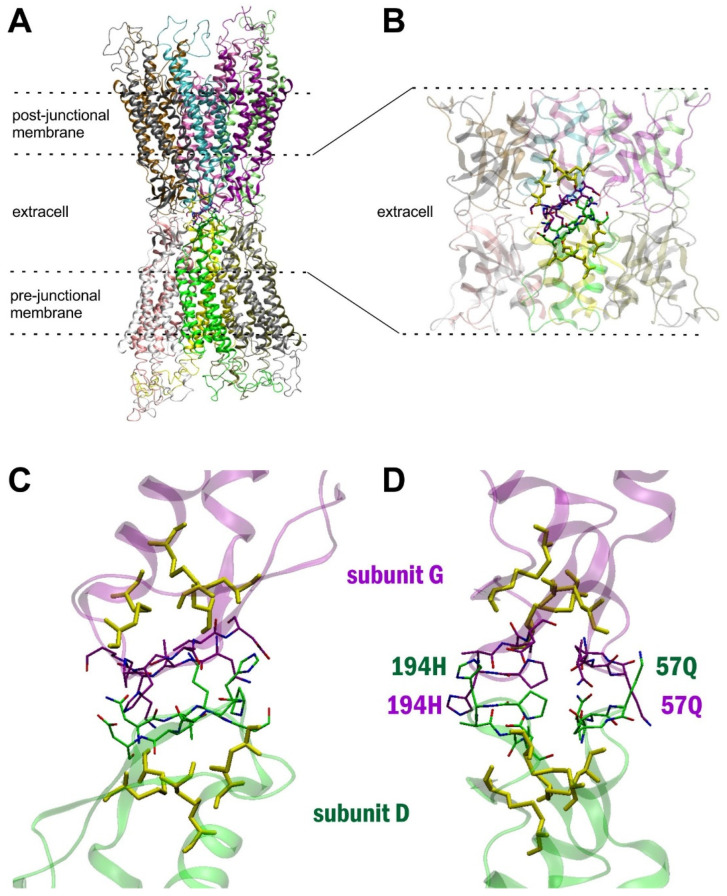
Structural design of amino acid residues filling the gap. (**A**): Full view of the Cx43 GJC homology model. Subunits are represented by different colours. (**B**): Extracellular loops of the Cx43 GJC homology model. Subunits are represented by different colours. Residues directly forming the GJ interface are shown in stick. (**C**,**D**): Mirror-apposition of pre-GJ “D” and post-GJ “G” subunits (*cf.* SI Figure S3) showing amino acid residues within 10 Å both from the conserved pre- and post-GJ cystine disulfide frontiers from inside (**C**) and side (**D**) views. Green and purple sticks represent the amino acid residues of each subunit within 10 Å from the conserved cystine disulfides, shown in yellow sticks. EL2 194H and EL1 57Q residues are highlighted, indicating outer and inner gap boundaries, respectively.

In Cx43 GJC model building, we used the X-ray structure of the homomeric Cx26 GJC dodecamer as a template (PDB code: 2zw3; SI Figure S1) [7,28] (*cf.* Methods). Our selection criteria were to provide high resolution data on residues participating in junctional coupling. As first described, the Cx26 X-Ray structure may well serve as a reference when connexon channel structures are compared. Initial inspection of the extracellular domain of the Cx43 GJC model [15] (*cf.* Methods) highlighted pre- and post-junctional cystine disulfide interfaces (Figure 1). In addition, certain Cys residues seem to form protein stabilization centers (SCs), meaning that although they are far in sequence, at least one of their atoms are closer than VWR(1) + VWR(2) + 1 Å, where VWR designates van der Waals radii (VWR) and residues in their vicinity also intensively interact with each other [29] (*cf.* SI Figure S2). These features prompted us to explore SCs potentially established between EL1 (47**D**-48**E**-49**Q**-50**S**-51A-52**F**-53R-54**C**-55**N**-56**T**-57Q-58**Q**-59**P**-60**G**-61**C**-62E-63**N**-64**V**-65**C-**66**Y**-67**D**-68K-69S-70**F**-71**P**-72**I**-73**S**) and EL2 (177**Y**-178**G**-179**F**-180S-181L-182S-183A-184V-185Y-186T-187**C**-188K-189R-190D-191**P**-192**C**-193**P**-194H-195Q-196**V**-197**D**-198**C**-199**F**-200L-201**S**-202**R**-203**P**) (residues conserved or partly conserved between the ELs are marked in bold).

**Figure 2:**
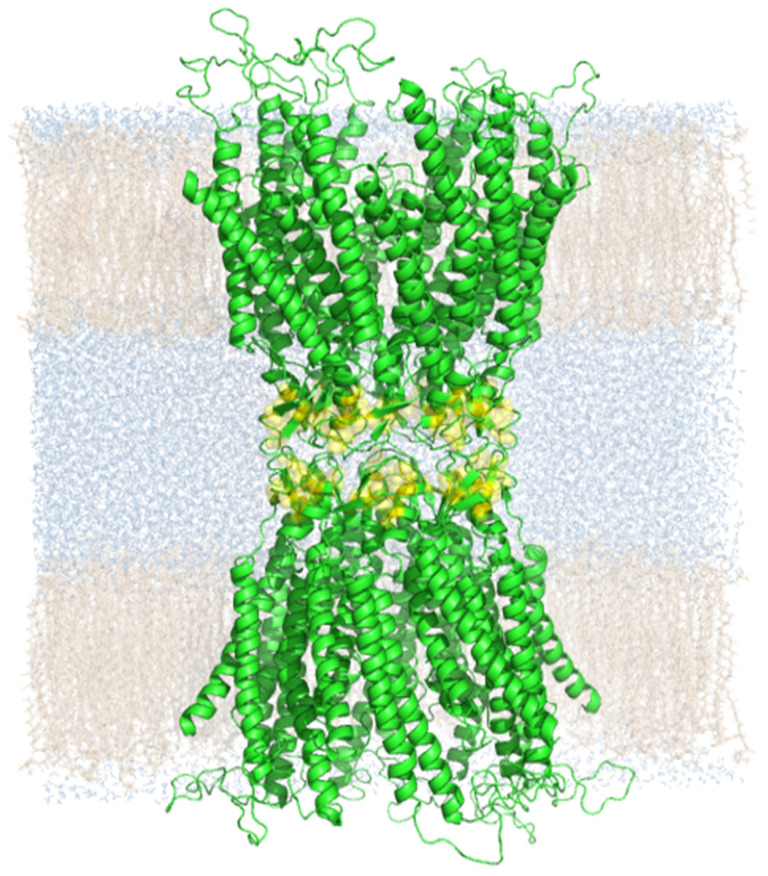
Side view of the Cx43 GJC model with two membrane bilayers after 100 ns standard MD with “close S-S bonds” precondition. Color code: grey-fatty acids in membrane bilayers; blue–water molecules; yellow–cystine disulfides.

The illustrated structural features of the GJ interface of the Cx43 GJC homology model prompted us to search for SCs in order to identify the regions responsible for building up the interface between the pre- and post-GJ Cx. To this end, we embedded the Cx43 GJC model in a double bilayer constituting the pre- and post-GJ membranes. To investigate the stability and dynamics of SC pairs, the Cx43 GJC model structure was allowed to fluctuate in its membrane environment in a 100 ns molecular dynamics (MD) simulation with standard *“close S-S bonds”* precondition, when the disulfide bonds were kept intact (Figure 2).

**Figure 3:**
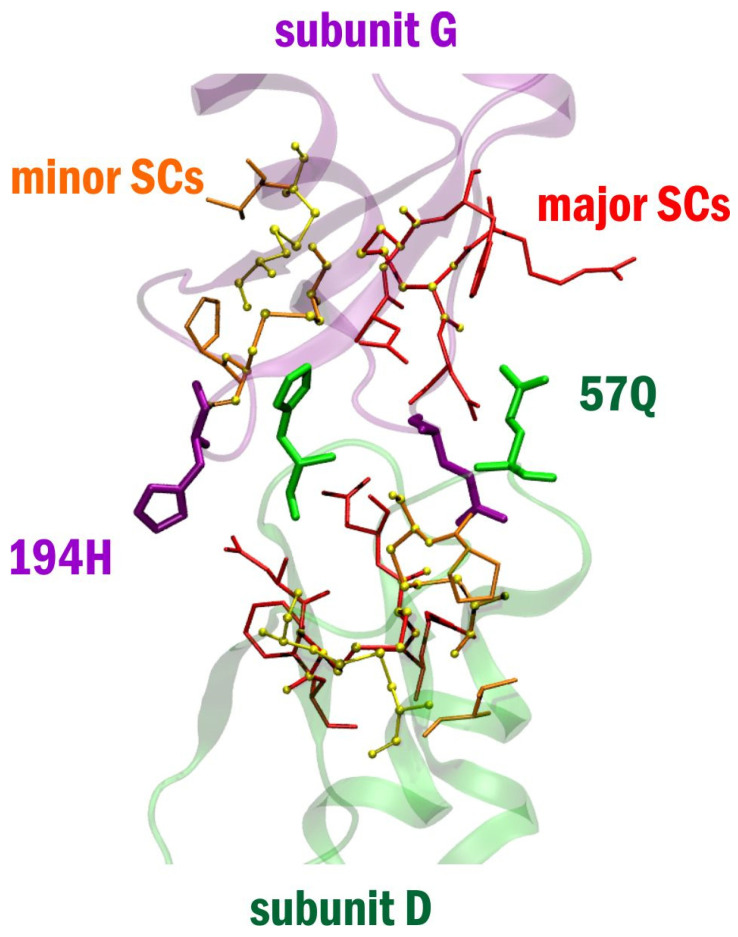
Inter-loop stabilization centers in the Cx43 GJ interface domain. Colour code: green cartoon—subunit D; purple cartoon—subunit G; major stabilization centers (53R, 54**C**, 55**N**, 197**D**, 198C, 199F) are shown in red thin stick, minor stabilization centers (61**C**, 64**V**, 191**P**, 192**C**) are shown in orange thin stick. Cystine disulfides are shown in thin yellow stick. Atoms of cystine disulfides are also marked by yellow ball to allow their visualization in case they are involved in stabilization centers as well. 57Q, 194H are shown in thick stick colored according to the subunit they belong to.

**Figure 4:**
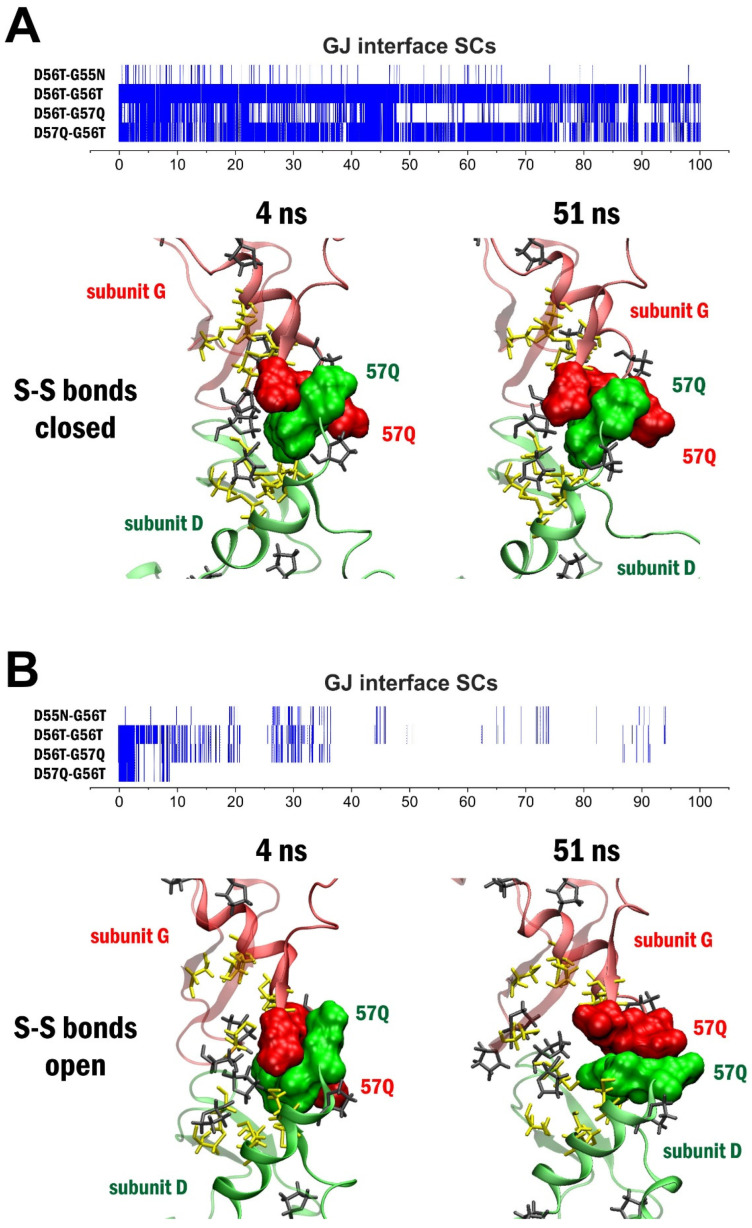
Forming of GJ interface SCs 55**N**-56**T**-57Q is governed by neighboring Cys residues. (**A**): Dynamics of GJ interface SCs between the D and G subunits during the closed disulfide MD run of Cx43 GJC (top). Bottom: 55**N**, 56**T** and 57Q residues (shown by surface representation) constitute the GJ interface stabilization centers (SCs) between the opposing subunits D (green) and G (red). The GJ interface SCs are fairly stable during the 100 ns molecular dynamics simulation. (**B**): Dynamics of GJ interface SCs between the D and G subunits during the open disulfide MD run of Cx43 GJC (top). Bottom: opening of S-S bonds at the beginning of the simulation leads to weakening of SC contact interactions between the 55**N**-56**T**-57Q surfaces and disruption of the SCs (bottom row). Color code: green–subunit D; red–subunit G; yellow–Cys residues; gray–Pro residues.

Under the “close S-S bonds” precondition, fluctuation dynamics of GJ interface SCs (SI Figure S4A) validated our initial observation that the GJ coupling of the two apposed connexons is mediated by the formation of a unique SC pattern with conserved (55**N**, 56**T**) and non-conserved (57Q) EL1 residues as it was postulated earlier [1]. It is worth noting that, despite each connexin in the pre-GJ interface contacts two connexins on the opposing site [1], SCs are selectively formed, with one of these opposing connexins (SI Figure S4A). As being the only one of its kind, a unique GJ interface SC contacting pre- and post-GJ 55**N**, 56**T** and 57Q along with cystine disulfides ^54**C**^S-S^198**C**^ has been disclosed (Figure 3 and Figure 4). No other GJ interface SC has appeared, suggesting that neighbouring cystine disulfide bond formation may trigger the formation of GJ interface SC contact and subsequent gap sealing.

In order to further our understanding of the structural prerequisites of the GJ interface formation, we next explored the SCs responsible for the stabilization of the EL1-EL2 interactions. Fluctuation dynamics of intra-subunit, inter-loop SCs (SI Figure S5) identified numerous SC patterns between EL1 and EL2. According to the occurrence and stability of these SC pairs, we classified two, spatially distinct groups of residues: the major SCs formed by 53R, 54**C**, 55**N**, 197**D**, 198**C**, and 199**F,** and the minor SCs, formed by 61**C**, 64**V**, 191**P**, and 192**C** (Figure 3). Besides, an intra-EL1 SC comprising the conserved 52**F** and 65**C** also emerges, possibly sustained by sulphur lone-pair and aromatic pi electron shuffling [30,31,32]. The participation of 54**C**, 61**C**, 65**C**, 192**C** and 198**C** residues in the SC pairs conclusively suggest that cystine disulfide S-S bonds play a significant role in the GJ interface formation. In the Cx43 GJC model with two membrane bilayers, the emergence of **^C^**S-S**^C^** linked GJ SC dynamics conjure up a decisive role for double membrane constraint played in shaping the GJ physique [3]. It is notable that the major SC pattern made up for non-conserved 57Q could also be assessed as a subtype-specific inhibitor template. Likewise, the minor SC pattern may get hold of subtype-specificity by linking non-conserved amino acids, such as, for example, 62E and/or 190D.

Since **^C^**S-S**^C^** exchange has been shown to be allowed at physiological temperature [33], we also investigated the SC pattern and GJ interface interactions after opening up the disulfide bonds at the beginning of the 100 ns-long MD simulation. Fluctuation dynamics of GJ interface SCs in the *“open S-S bonds”* precondition showed reduced GJ interface SC coupling via residues 55**N**, 56**T** and 57Q (SI Figure S4B). Explicitly, instead of maintaining the twin half-rings form of the GJ interface SC motif related to the *“close S-S bonds”* precondition (Figure 4A), interaction between the 55**N**, 56**T** and 57Q residues have significantly weakened in the “*open* *S-S bonds”* precondition (Figure 4B). Disappearance of the unique GJ interface SC pattern due to disulfide bond openings, therefore, suggests that coupling of Cx43 connexons may depend on neighbouring cystine disulfide exchange. It is noteworthy in this regard that the Cx31.3 subtypes, in which 55N is mutated to 55H does not form functional GJC [5].

**Figure 5:**
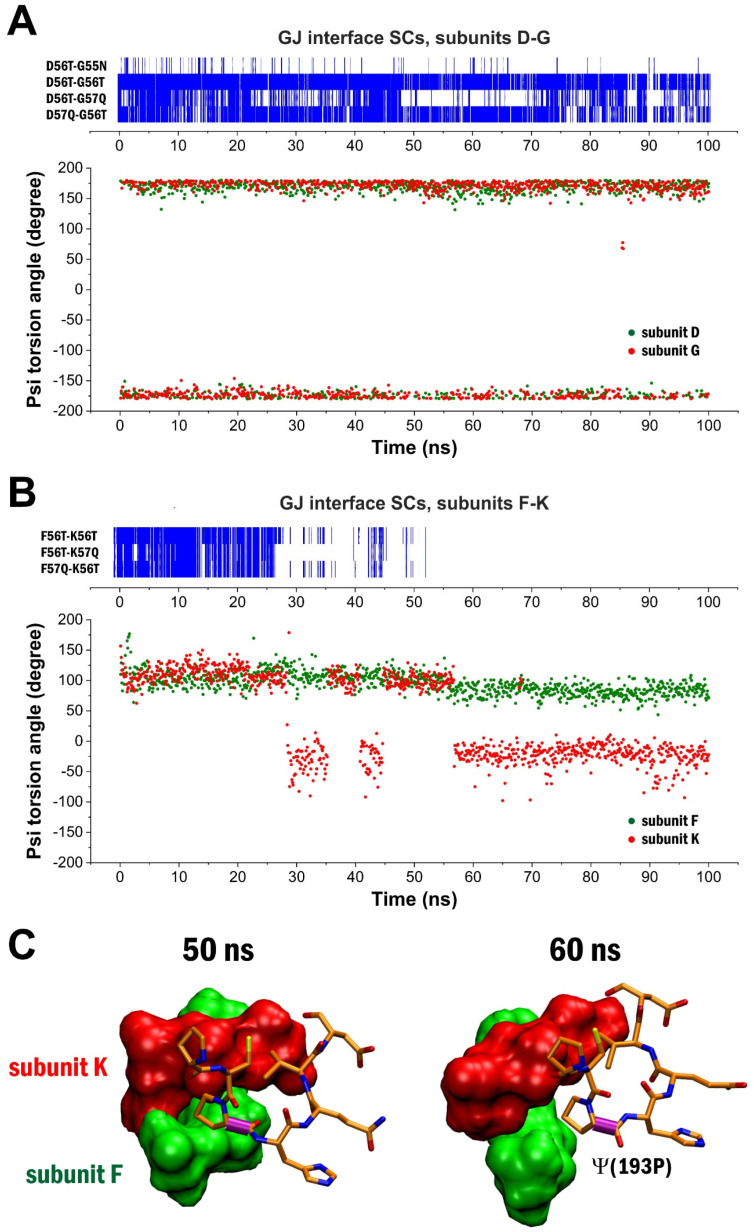
Appearance of GJ interface SCs coupling of opposing connexons by 55**N**-56**T**-57Q correlates with the ψ torsion angle of the nearby 193**P** residue. (**A**) Dynamics of GJ interface SCs between the D and G subunits during the closed disulfide MD run of Cx43 GJC (top) and the changes in the ψ (psi) torsion angle of 193**P** in both subunits. (**B**) Dynamics of GJ interface SCs between the F and K subunits during the closed disulfide MD run of Cx43 GJC (top) and the changes in the ψ (psi) torsion angle of 193**P** in both subunits. (**C**) 55**N**, 56**T** and 57Q residues (shown by surface representation) constitute the GJ interface stabilization centers (SCs) between the opposing subunits F (green) and K (red). The GJ interface SCs disappear in conjunction with the reorientation of the interface shaped by the 191–197 residues of the EL2 of subunit K. Color code: green–subunit F; red–subunit K. The ψ torsion angle of the 193**P** residue of subunit F is highlighted by a purple tube.

Besides the dynamic openings of disulfide bonds, the high number of proline residues in the close vicinity of extracellular cysteines (59**P**, 191**P** and 193**P**) also provide an opportunity to induce local conformation changes [34]. To explore the possibility that conformational flexibility of the proline residues may contribute to the appearance or disappearance of GJ interface SCs, we first measured the ω (omega) dihedral angles of 59**P**, 191**P** and 193**P** in the closed disulfide configuration. These angles were found to be confined to 180°, indicating the stable presence of the *trans-*proline configuration. However, ψ (psi) torsion angles were observed to be largely fluctuating in all subunits. Moreover, we found that the values of ψ torsion angles of the 193**P** residues significantly correlated to the presence of GJ interface SCs (Figure 5A,B). When the ψ torsion angle of 193**P** in either of the opposing connexin subunits moved to the range 0° to −45°, the GJ interface SCs built from 55**N**, 56**T** and 57Q have disappeared (Figure 5B). The high impact of 193**P** ψ torsion angle on the appearance of GJ interface SCs is likely attributed to the modified orientation of the 55**N**, 56**T** and 57Q interface due to EL1-EL2 interactions (Figure 5C). In the open disulfide configuration, we also observed changes in the EL1-EL2 interactions and corresponding fluctuations of the presence of GJ interface SCs due to changes in the ψ torsion angle of 193**P**, however, weakening of GJ interface SCs was also assigned to the increased distance between EL1 and EL2 in some subunits.

**Figure 6:**
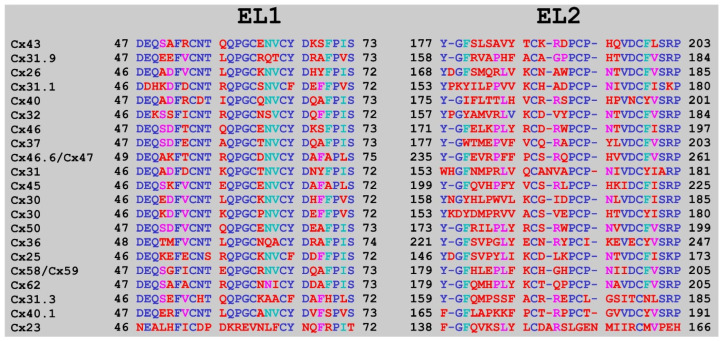
Alignments of extracellular cysteine-rich domain sequences of 21 Cx family members**.** Left: EL1; Right: EL2. The alignment has been calculated by Clustal [35] implemented in the Jalview multiple sequence alignment program [36]. Letters are colored according to the percentage of the residues in each column that agree with the consensus sequence (https://www.jalview.org/help/html/colourSchemes/pid.html). Only the residues that agree with the consensus residue for each column are colored. Letter code: DARK BLUE: > 80%; PALE BLUE: > 60%; MAGENTA: > 40%; RED: < 40%.

The inner EL1 features few non-conserved residues linking several conserved sequence-motifs (Figure 6). The encoded arrangement suggests a designer role for EL1 in connexon coupling and GJC pore formation. When associated via the cystine disulfide linkage, the less-conserved outer EL2 (Figure 6) can further contribute to junctional subtype-specificity. These considerations also justify the search for finding Cx subtype-specific inhibitors interfering connexons coupling to Cx GJC.

## 3. Discussion

Despite valuable insights into the structure and regulation of Cx HCs and GJCs [1,3,37,38,39,40,41,42,43,44,45,46,47,48,49,50,51,52,53,54,55,56,57], one may have the impression that matters arising from the lack of Cx subtype-specific inhibitors and pertinent structural issues stayed somewhat unexplored [58,59].

Taking cysteine-rich domains as major players of intercellular communication, we could refer to the cytoplasmic carboxy terminus securing Cx GJCs in plaques [60]. Much less is known about the functioning of extracellular cysteine-rich Cx domains. Importantly, extracellular loops have been proposed to act as Cx redox sensors and therapeutic targets [61,62]. Here we conjecture **^C^**S-S**^C^** disulfide bond exchange and the formation of the unique GJ interface SCs, contacting pre- and post-GJ 55**N**, 56**T** and 57Q residues. The emergence of **^C^**S-S**^C^** exchange in fluctuation dynamics of SCs may be relevant at factual cysteine/cystine redox potential values [63,64]. Besides, the exchange kinetics would be facilitated by electron flow [65] or tunnelling [66,67] through short Cα distances featuring GJ interfaces. Indeed, Cys-containing SCs seem to appear fast in the Cx43 GJC prototype, meaning a few nanoseconds or less.

Initially, cysteine-rich extracellular loop shaped anti-parallel beta sheets of pre- and post-GJ connexons were hypothesized to intermingle [14]. Based on Cx GJC simulations performed with close/open disulfide preconditions, however, they appear to explain the rather layered stacking of the anti-parallel beta sheets. Together with the unique GJ interface SC pattern formation, the novel gap building paradigm gives the Cx subtype specific inhibition a chance, still awaited [15,68,69,70,71,72]. In order to design Cx subtype-specific inhibitors of connexon-connexon coupling, we propose to mimic GJ structure-motifs comprising non-conserved SC-forming residues. It may have relevance in that the EL2 residue 188N of human Cx46 plays a critical role in connexon coupling related to the cataract associated mutation N188T [73]. The perspective of designing small molecules mimicking subtype-specific structural motifs in the GJ domain of GJCs shall serve the discovery and development of more specific Cx GJC inhibitors in the future.

In order to approach real world relevance in our results, we should take into account that cardiomyocytes [21,23,74,75] and astrocytes (SI Table S1) are heavily interconnected by GJCs involving both homomeric and heteromeric Cx43 connexons, raising matters of vital side-effects of potentially Cx43 subtype-specific therapeutics. We may see the point by relating cellular/sub-cellular appearances of Cx43 connexons and interactions between glia and neurons modulating neuronal signalling on local/longer spatial scales by way of Glu, Ca^2+^ and K^+^ signalling, spreading synchronization [76,77,78,79,80] and distributing metabolic energy supply [81]. Brain area-dependent cellular allocations of heteromeric Cx43 connexons appear to relate distinguishable Cx43 functioning at astrocyte-neuron (Cx43 with Cx26, Cx30, Cx30.2, Cx36, Cx45, Cx32) and astrocyte-oligodendrocyte (Cx43 with Cx47) interfaces (SI Table S1). Meaningfully, the visual, parietal and frontal cortical structures express homomeric Cx43 connexons only (SI Table S1) [82], invoking key function of homomeric Cx43 GJCs during both slow wave and paroxysmal activities in the neocortex [25,26,27,83]. Earlier, gap junction blockers were shown to suppress seizure-like activities both in vitro [84,85,86,87] and in vivo [88,89]. As outlined above, the high incidence of homomeric Cx43 GJC signalling in slow wave and paroxysmal activities in the neocortex, however, necessitates the exploration of Cx43 GJC subtype-specific structural motifs in the GJ target domain for future drug discovery campaigns.

We may distinguish domains of Cx GJC functioning. As mentioned before, the cysteine-rich carboxy terminal (CT) determines plaque stability [53]. It may have relevance in the context that the intrinsically disordered CT region binds with Cx43 interaction protein of 75 kDa (CIP75) that appears to regulate the degradation of Cx43 along with Cx40 and Cx45 GJCs [74,75]. Data and simulations suggest that channel properties of Cx46/Cx50 GJCs are affected by amino terminal (NT) and EL1 domain residues, probably lining the pore [90,91,92,93]. These experiences face GJCs at work, however, much less is known about the connexon coupling to GJC. Our simulations show that the cysteine-rich extracellular loops control connexon-connexon coupling via the formation of Cx43 subtype-specific GJ structures. These findings open up the possibility to invalidate coupling and to abort GJC function this way by devising inhibitors that mimic Cx43-specific protein stabilization centers.

## 4. Methods

### 4.1. Preparation of the Cx43 GJC Homology Model

*Building Cx26-based homology model of Cx43 GJC.* Cx43 GJC was built up as described previously [15]. In short, we used the automated mode of the Swiss-Model server [94], based on the X-ray structure of the Cx26 GJC (PDB code: 2zw3) [28]. Technically, the primary sequence of the human Cx43 protein (Uniprot code: P17302) was submitted to the website of Swiss-Model, which offered the homologous 2zw3 structure as a template (https://swissmodel.expasy.org/interactive, accessed on 1 July 2018). Notably, the Cx26 GJC lacks the long and disordered CT domain appearing in the Cx43 GJC. Then, Swiss-Model generated an alignment between the target and the template (SI Figure S1) and built the 2zw3 structure-based model. During model building the backbone coordinates of the well aligned regions were taken directly from the template, while insertions and deletions were remodelled using a fragment library, and finally side-chains were modelled, followed by a short energy minimization. Our initial model contained 12 subunits: A to F for one Cx43 HC and G to L for the opposing Cx43 HC (SI Figure S3). This initial Cx43 GJC model was submitted to the “Positioning Proteins in Membrane” (PPM) server of the “Orientations of the Membranes in Proteins” (OPM) database [95] to predict Cx43 trans-membrane regions 21–46, 74–93, 156–176, 204–230 [15].

### 4.2. Preparation of the Cx43 GJC Homology Model with Two Membranes Using Virtual Molecular Dynamics (VMD)

For the reason that VMD allows the application of more than one membrane per protein, we prepared Cx43 GJC structures using the VMD 1.9.3 tutorial [96] as described [7]. In short, the VMD process (http://www.ks.uiuc.edu/Training/Tutorials/science/membrane/mem-tutorial.pdf, accessed on 10 July 2019) requires coordinate files (PDB) and structure files (psf) for all types of calculations. While PDB files are used generally, the psf files are specific to VMD, comprising names of atoms, coordinates, capping residues for N and C termini, topology files for lipids and proteins and others, such as the specification of cystine disulfide bond residues. First, to generate the psf file, the Cx43 GJC dodecamer model should be disconnected into the twelve individual subunits as the “psfgen” program expects non-covalently linked segments being in separate pdb files. In order to accomplish the condition of 100 ns MD of Cx43 GJC at 300 K with “*close S-S bonds*” precondition, protein subunits were prepared by setting disulfide bonds between residues 54C-198C, 61C-192C and 65C-187C in the psf file of each subunit. For the condition of the Cx43 GJC (100 ns) at 300 K with the “open *S-S* bonds” precondition, or the “partially open S-S bonds” precondition, where 61C-192C was opened, the Cx43 GJC subunits were also prepared individually, but without setting the S-S bonds. For the wild type runs we started from the structure obtained from OPM (see previous section), while for the mutant N55H, D197T we introduced the mutations in the OPM generated structure by Pymol. Subsequently, we followed the same protocol for both Cx43 GJC structures with and without setting extracellular S-S bonds. The 12 subunits were re-combined into a single file once again and the “Membrane builder plugin” of VMD was used to generate two POPC membranes (150 × 150 Å each). Membranes were aligned manually relative to the protein by translation along with “z” direction, leaving the extracellular E1 and E2 loops (47–73 and 177–203, respectively) membrane-free. In order to handle two different membranes, segment IDs of the lipid residues (P11, P21 *etc.*) and water (W1, W2 *etc.*) are to be renamed before switching over the second membrane. After combining the two membranes with the protein we used the CHARMM 27-derived [97] “top_all27_prot_lipid.inp” topology file for protein-lipid systems. Topology files contain the type, mass, charge and connectivity of every atom together with internal coordinates that help to define the position of hydrogen atoms. Next, “bad water and bad lipid” molecules overlapping with the protein (terminology according to the tutorial) were removed, and lipids were also cut out from the channel within the 10 Å radius from the center. To generate water box we used VMD’s solvate package. The system was ionized with Na^+^ and Cl^−^ to a physiological ionic concentration of 0.15 mol/L using the “Autoionize module” of VMD. Thereafter, the system was ready for minimization and MD running.

MD runs were performed as described [7] applying NAMD 2.12 with CUDA using the CHARMM27 force-field [97] in four steps. During the first step, only lipid chains were allowed to move at 300 K for 0.5 ns (also called „melting of lipid chains”). Secondly, water molecules were allowed to move at 300 K for 0.5 ns, while the protein was still held fixed. Then, restraints were released on the protein and the entire system was equilibrated for 0.5 ns to arrange the lipid and water around the protein. Finally, we employed the all-atom production MD run for 100 ns according to the VMD protocol implying Langevin dynamics at constant temperature of 300 K along with the constant pressure NPT condition of 1 atm. As already given formerly, MD data were assessed by 2 fs time-steps and 10 ps sampling time.

### 4.3. SC Search and Analysis

In order to identify stabilization centers (SCs), MD trajectories from NAMD or Desmond were imported into VMD and individual frames at 10 ps interval were exported as .pdb files. After adding sequence residues (SEQRES) data by Maestro, SCs were identified in these .pdb files by the stabilizing residues (SRide) server [98]. Extracellular SCs were filtered by selecting those SCs, containing at least one extracellular amino acid (corresponding to residues 47–73 or 177–203). These SCs were further filtered for frequent SCs appearing in at least 2% of the total running time.

### 4.4. Dihedral Angle Measurements

Phi and psi dihedral angles for proline residues 59P, 191P and 193P were calculated by VMD. The omega angle for the residue on the N-terminal side of these prolines were also calculated, since rotation around this C-N bond may induce major structural changes [34].

### 4.5. Alignment Calculations

The Uniprot database was searched for keywords “name: gap junction” and “organism: human”, which resulted in 21 human Cx subtypes. These sequences were downloaded to a common fasta file and opened in Jalview [36]. The alignment was calculated with Clustal [35] using default values. Cx43 was set as reference. Subsequently, the EL1 and EL2 regions were selected and coloured according to percent identity.

## Data Availability

The data presented in this study are available in the main text, figures, tables and supplementary material.

## Supplementary Materials

The following supporting information can be downloaded at: https://www.mdpi.com/article/10.3390/biom12010049/s1, Figure S1: Sequence alignment between Cx43 and Cx26, Figure S2: Schematic representation of stabilization center (SC) elements, Figure S3: Side view of the dodecameric GJC assembly of Cx43 subunits, Figure S4: Fluctuation dynamics of trans-junctional stabilization centers (SCs) of Cx43 GJC model, embedded in two membranes, Figure S5: Fluctuation dynamics of intra-subunit, inter-loop stabilization centers (SCs) of Cx43 GJC model, embedded in two membranes. Table S1: Anatomical distribution of Cx43 GJC versus Cx36 GJC subtypes in the brain.
