# Supplementary material for: Connexons Coupling to Gap Junction Channel: Potential Role for Extracellular Protein Stabilization Centers
Source: Biomolecules. 2021 Dec 30;12(1):49. doi: 10.3390/biom12010049 (PMC8773650; doi:10.3390/biom12010049)
Supplement: Supplementary file 1 [file biomolecules-12-00049-s001.zip › biomolecules-1495992-supplementary.pdf]

Connexons Coupling to Gap Junction Channel: Potential Role for Extracellular Protein Stabilization Centers

László Héja*, Ágnes Simon, Zsolt Szabó and Julianna Kardos

Institute of Organic Chemistry, Research Centre for Natural Sciences, Magyar tudósok körútja 2, 1117, Budapest, Hungary.

Supplementary Information (SI)

SI Figures

SI Figure S1. Sequence alignment between Cx43 and Cx26.

SI Figure S2. Schematic representation of stabilization center (SC) elements.

SI Figure S3. Side view of the dodecameric GJC assembly of Cx43 subunits.

SI Figure S4. Fluctuation dynamics of trans-junctional stabilization centres (SCs) of Cx43 GJC model, embedded in two membranes.

SI Figure S5. Fluctuation dynamics of intra-subunit, inter-loop stabilization centres (SCs) of Cx43 GJC model, embedded in two membranes.

SI Tables

SI Table S1. Anatomical distribution of Cx43 GJC versus Cx36 GJC subtypes in the brain.

SI References

```
Cx43    1  MGDWSALGKLLDKVQAYSTAGGKVWLSVLFIFRILLLGTAVESAWGDEQSAFRCNTQQPGCENVCYDKSFPISHVRFWVL   80
Cx26    1  -MDWGTLQTILGGVNKHSTSIGKIWLTVLFIFRIMILVVAAKEVWGDEQADFVCNTLQPGCKNVCYDHYFPISHIRLWAL   79

Cx43   81  QIIFVSVPTLLYLAHVFYVMRKEEKLNKKEEELKVAQTDGVNVDMHLKQIEIKKFKYGIEEHGKVKMRGGLLRTYIISIL  160
Cx26   80  QLIFVSTPALLVAMHVAYRRHEKKRKFIKGEIK---------SEF--KDIE--EI-----KTQKVRIEGSLWWTYTSSIF  141

Cx43  161  FKSIFEVAFLLIQWYIY-GFSLSAVYTCKRDPCPHQVDCFLSRPTEKTIFIIFMLVVSLVSLALNIIELFYVFFKGVKDR  239
Cx26  142  FRVIFEAAFMYVFYVMYDGFSMQRLVKCNAWPCPNTVDCFVSRPTEKTVFTVFMIAVSGICILLNVTELCYLLIRYCSGK  221

Cx43  240  VKGKSDPYHATSGALSPAKDCGSQKYAYFNGCSSPTAPLSPMSPPGYKLVTGDRNNSSCRNYNKQASEQNWANYSAEQNR  319
Cx26  222  SKKP----------------------------------------------------------------------------

Cx43  320  MGQAGSTISNSHAQPFDFPDDNQNSKKLAAGHELQPLAIVDQRPSSRASSRASSRPRPDDLEI
Cx26       ---------------------------------------------------------------
```

SI Figure S1. Alignment between Cx43 and Cx26 [Simon et al., 2020]. Sequence alignment between Cx43 and Cx26 (PDB code: 2zw3) [Maeda et al., 2009] generated by Swiss-Model show high sequence similarity. It appears in the extracellular domain and at transmembrane (TM) regions between TM1 and TM2 along with TM3 and TM4 regions. Insertions and deletions are found only in the intracellular regions. Amino acids (AA) of Cx43 and Cx26 TM regions are shown in red.

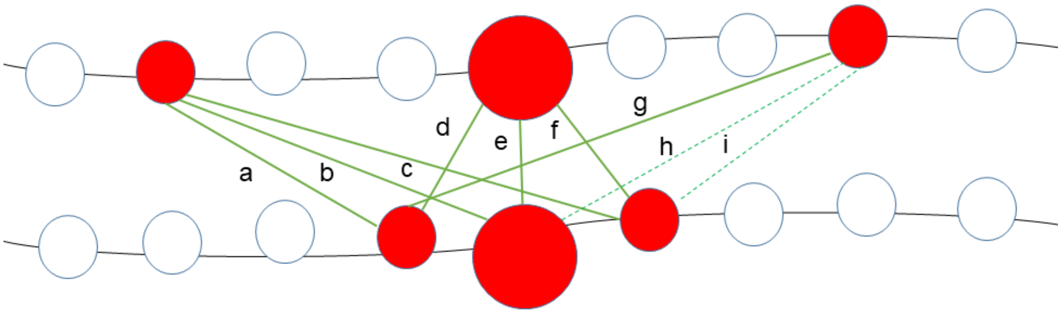

SI Figure S2. **Schematic representation of stabilization center (SC) elements** *(modified after* [Nyikos et al., 2002; Simon et al., 2001]). The central amino acid residues (AAs) are indicated by large red circles. In addition, small circles on each side (*flanking tetrapeptides*) comprise the supporting AAs filled in red. These three red AAs on each chain (*triplets*) form potential contacts (lines "*a-i*"). The central residues form an SC if they are at least 10 residues away in sequence, and it is possible to select two neighbours from their *flanking tetrapeptides*, so that the resulting two *triplets* form 7 contacts ("*a-g*", green solid lines). Two missing contacts "*h-i*" are indicated by green dots [Dosztányi et al., 1997]. Two AAs are considered to be in contact if at least one of their atoms are closer than VWR(1) + VWR(2) + 1 (Å), where VWR designates van der Waals radii (VWR). *For a list of atomic VWRs see:* https://github.com/evocellnet/int3dInterfaces/blob/master/vdw.radii

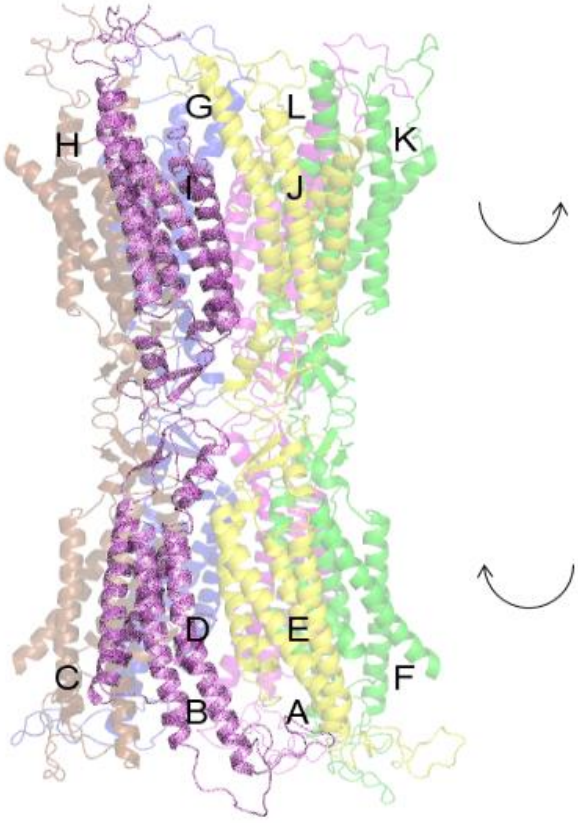

SI Figure S3. Side view of the dodecameric GJC assembly of Cx43 subunits. After Maeda et al. [2009]. Both Cx43HC contain six subunits that are coloured as follows: A & J: yellow, B & I: violet (highlighted), C & H: brown, D & G: blue, E & L: magenta, F & K: green. Note, that the subunits of HC_{pre} and HC_{post} follow counter-clockwise and clockwise directions, respectively.

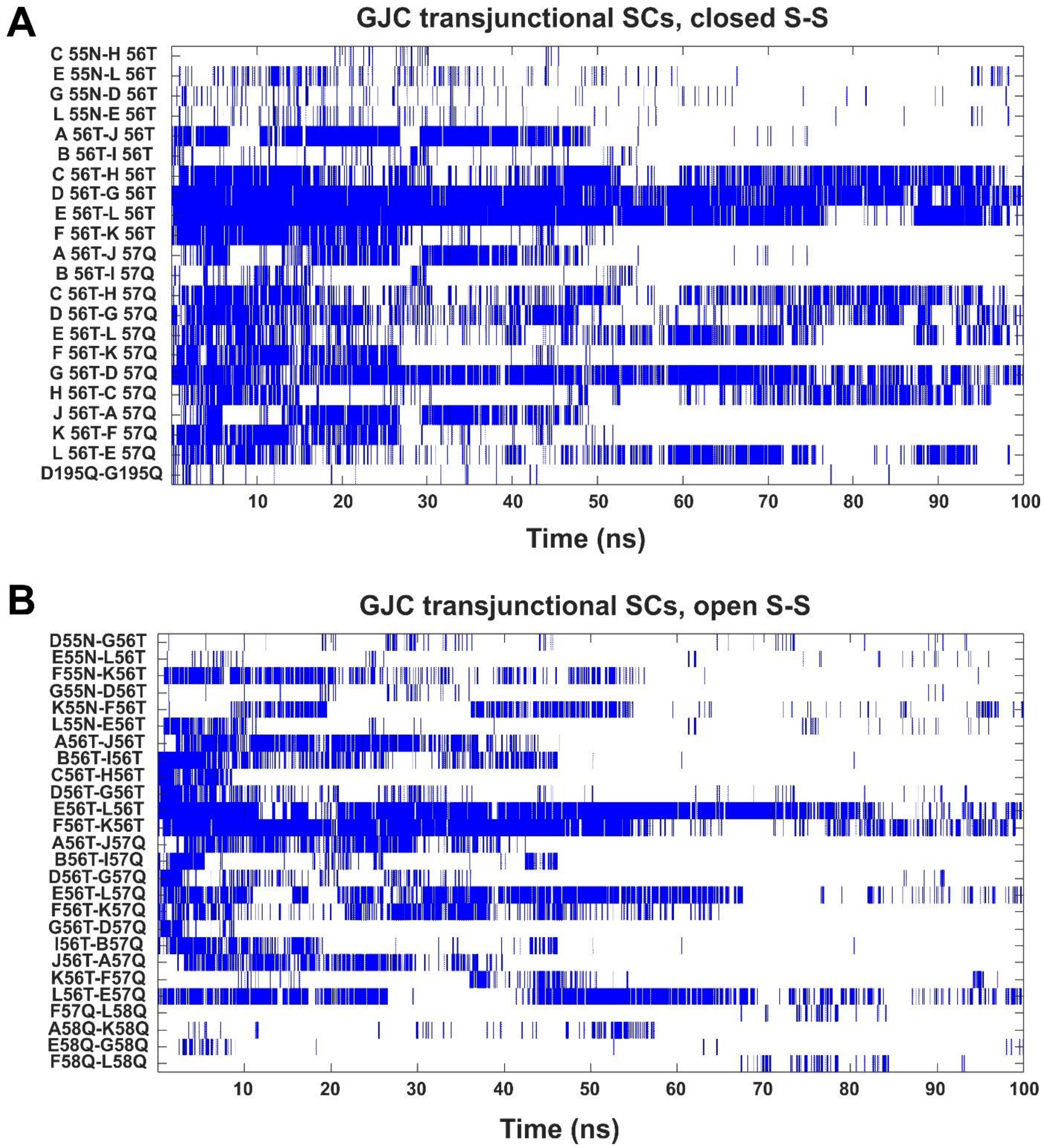

SI Figure S4. **Fluctuation dynamics of trans-junctional stabilization centres (SCs) of Cx43 GJC model, embedded in two membranes.** 100 ns MD simulation was performed at 300 K. S-S bonds were kept intact during the MD simulation (**A**) or have been opened up at the start of the MD simulation (**B**). Each line corresponds to a single SC pair. The residues forming a single SC pair are displayed as the y axis. First letter of each residue represents the subunit (A to F corresponds to the pre-junctional, G to L corresponds to the post-junctional subunits). Only SC pairs formed between the opposing connexons and appearing at least 1 % of the simulation are displayed.

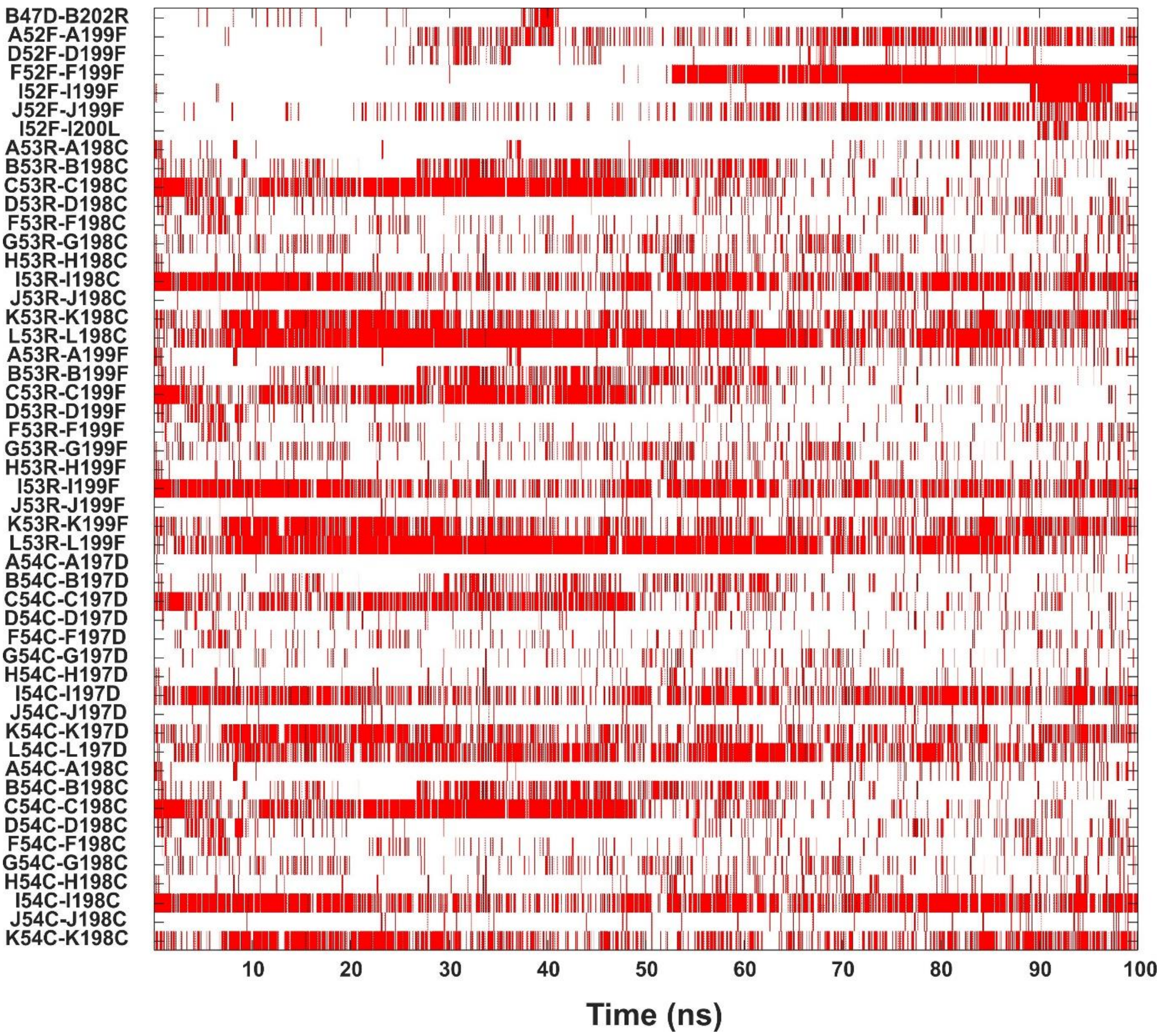
B47D-B202R
A52F-A199F
D52F-D199F
F52F-F199F
I52F-I199F
J52F-J199F
I52F-I200L
A53R-A198C
B53R-B198C
C53R-C198C
D53R-D198C
F53R-F198C
G53R-G198C
H53R-H198C
I53R-I198C
J53R-J198C
K53R-K198C
L53R-L198C
A53R-A199F
B53R-B199F
C53R-C199F
D53R-D199F
F53R-F199F
G53R-G199F
H53R-H199F
I53R-I199F
J53R-J199F
K53R-K199F
L53R-L199F
A54C-A197D
B54C-B197D
C54C-C197D
D54C-D197D
F54C-F197D
G54C-G197D
H54C-H197D
I54C-I197D
J54C-J197D
K54C-K197D
L54C-L197D
A54C-A198C
B54C-B198C
C54C-C198C
D54C-D198C
F54C-F198C
G54C-G198C
H54C-H198C
I54C-I198C
J54C-J198C
K54C-K198C
10 20 30 40 50 60 70 80 90 100
Time (ns)

(cont.)

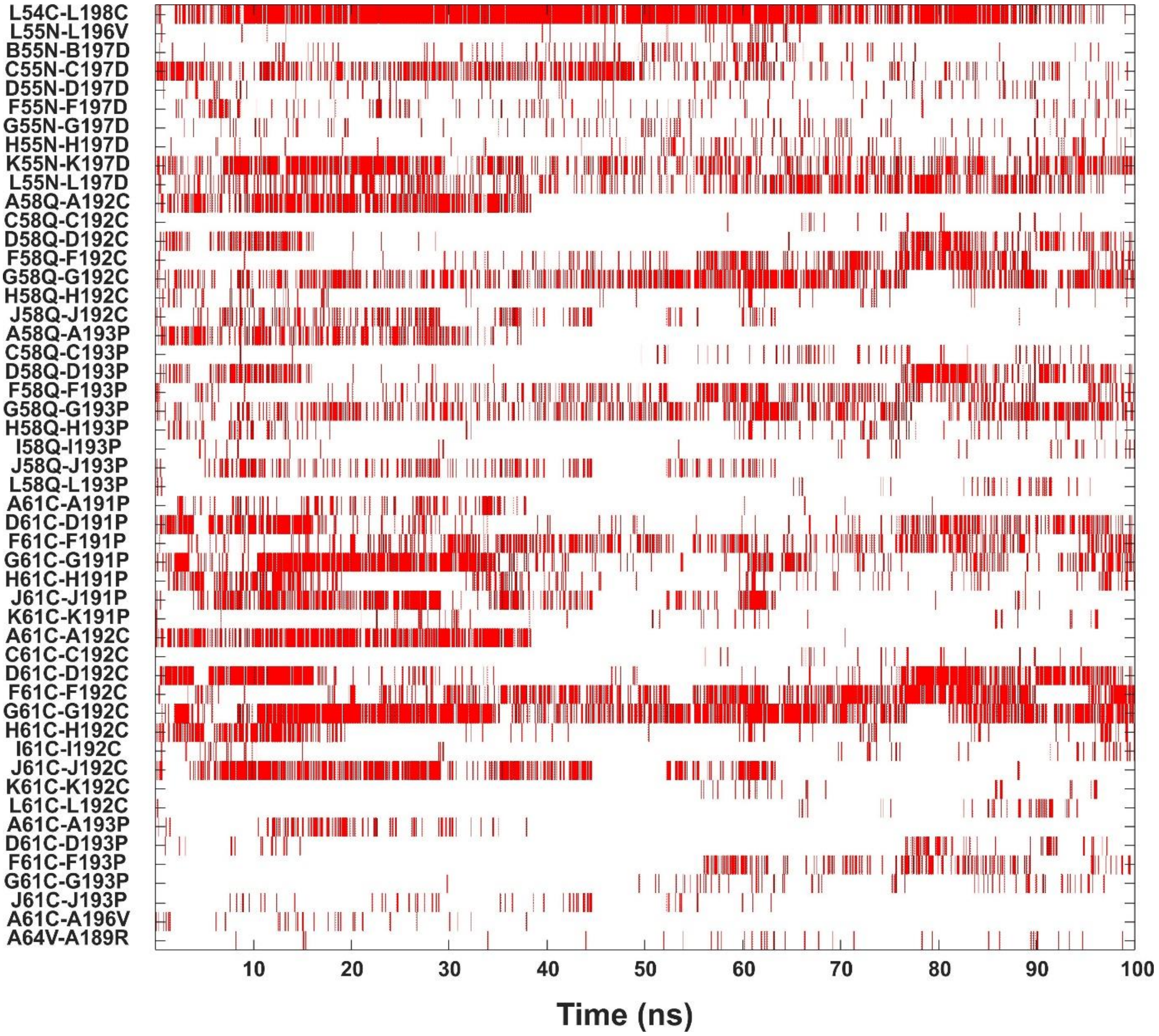
L54C-L198C
L55N-L196V
B55N-B197D
C55N-C197D
D55N-D197D
F55N-F197D
G55N-G197D
H55N-H197D
K55N-K197D
L55N-L197D
A58Q-A192C
C58Q-C192C
D58Q-D192C
F58Q-F192C
G58Q-G192C
H58Q-H192C
J58Q-J192C
A58Q-A193P
C58Q-C193P
D58Q-D193P
F58Q-F193P
G58Q-G193P
H58Q-H193P
I58Q-I193P
J58Q-J193P
L58Q-L193P
A61C-A191P
D61C-D191P
F61C-F191P
G61C-G191P
H61C-H191P
J61C-J191P
K61C-K191P
A61C-A192C
C61C-C192C
D61C-D192C
F61C-F192C
G61C-G192C
H61C-H192C
I61C-I192C
J61C-J192C
K61C-K192C
L61C-L192C
A61C-A193P
D61C-D193P
F61C-F193P
G61C-G193P
J61C-J193P
A61C-A196V
A64V-A189R
10
20
30
40
50
60
70
80
90
100
Time (ns)

(cont.)

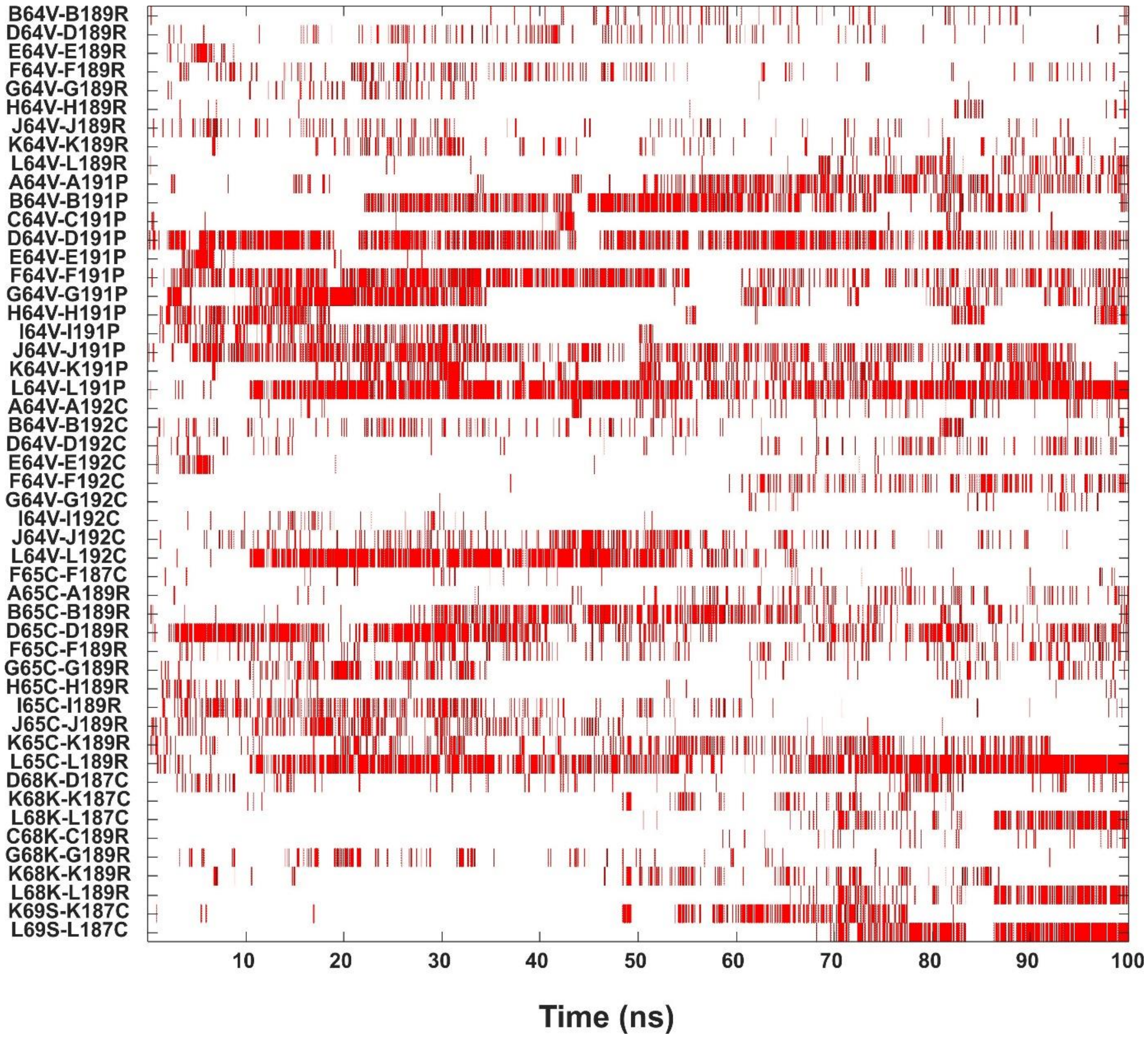

SI Figure S5. **Fluctuation dynamics of intra-subunit, inter-loop stabilization centres (SCs) of Cx43 GJC model, embedded in two membranes.** 100 ns MD simulation was performed at 300 K. S-S bonds were kept intact during the MD simulation. Each line corresponds to a single SC pair. The residues forming a single SC pair are displayed as the y axis. First letter of each residue represents the subunit (A to F corresponds to the pre-junctional, G to L corresponds to the post-junctional subunits). Only SC pairs formed between the EL1 and EL2 loops of the same subunit and appearing at least 1 % of the simulation are displayed.

SI Tables

SI Table S1. Functional anatomy and distribution of Cx43 GJC versus Cx36 GJC subtypes in the brain. Connexins are expressed in mixed electrical and chemical synapses, purely electrical synapses, astrocyte-neuron and astrocytes-oligodendrocyte GJCs.

Mixed electrical & chemical synapses

Connexin types	Tissue	Structure	Method	Refs.	GJ nomenclature used in the study
unknown	HC<br>CA3 *stratum lucidum*	hippocampal mossy fibers (MFs) & pyramidal cells	Patch-clamp	Vivar et al. 2012	Gap junction
Cx36	Ventral HC<br>CA3 *stratum lucidum*	MF terminals on pyramidal neuron thorny excrescences (Glutamatergic)	IF	Nagy 2012	Gap junction<br>/gap junction plaques
Cx36	HC<br>CA3 *stratum lucidum*	MF terminals on pyramidal neuron thorny excrescences (Glutamatergic)	EM, patch-clamp	Münster-Wandowski et al. 2013	Gap junction
Cx36	HC<br>CA3 *stratum lucidum*	MF terminals on pyramidal neuron thorny excrescences (Glutamatergic)	EM	Hamzei-Sichani et al. 2012	Gap junction/<br>reticular gap junction
Cx36	HC<br>CA3 pyramidal neurons	GABAergic (rare)	EM	Hamzei-Sichani et al. 2012	Gap junction/<br>reticular gap junction - previously called “anastomosing” gap junctions
Cx36	HC<br>CA3 *stratum oriens*	spiny interneuron (Glutamatergic)	EM	Hamzei-Sichani et al. 2012	Gap junction
Cx36	HC<br>Dentate gyrus	dendritic spines of interneurons & unidentified neurons	EM	Hamzei-Sichani et al. 2012	Gap junction
Cx36	HC<br>Hilus	dendritic spines of interneurons & unidentified neurons	EM	Hamzei-Sichani et al. 2012	Gap junction/<br>reticular gap junction - previously called “anastomosing” gap junctions
Cx36	Spinal cord	motor nuclei of lamina IX	IF	Bautista et al. 2014, Bautista & Nagy, 2014	Gap junction
Cx36	Spinal cord	laminae V-V1, medial lamina	IF	Bautista et al. 2014,	Gap junction

		VII , Clarke's nucleus		Bautista & Nagy, 2014	
Cx36	Trigeminal motor nucleus (Mo5)	peripherin-positive motoneurons	IF	Bautista et al. 2014, Bautista & Nagy, 2014	Gap junction
Cx36	Midbrain	red nucleus	IF	Nagy et al. 2019	Gap junction
Cx36	Auditory system	ventral cochlear nucleus	IF	Rubio & Nagy 2015	Gap junction/ gap junction plaque
Cx36	Auditory system	Medial nucleus of the trapezoid body	IF	Rubio & Nagy 2015	Gap junction/ gap junction plaque
Cx36	Auditory system	Lateral superior olivary complex	IF	Rubio & Nagy 2015	Gap junction/ gap junction plaque
Cx36	Olfactory bulb	Mitral cells	EM, patch-clamp	Christie et al. 2005	Gap junction
Cx36	Olfactory bulb	Mitral/tufted cell dendrites	EM	Kosaka & Kosaka 2005; Kosaka et al. 2005	Gap junction
Cx36, and less abundantly Cx45	Olfactory bulb	Mitral/tufted cells	EM, IF	Rash et al. 2005	Gap junction
Cx36	Brainstem	Lateral vestibular nucleus	IF	Nagy et al. 2013	Gap junction/ gap junction plaque
Cx57	Retina	B-type horizontal cells	EM, IF	Pan et al. 2012, Dorgau et al. 2015	Gap junction ("classical pentilaminar appearance not present")

Purely electrical synapses

Cx36	CA3 Stratum oriens	Interneuronal dendro-dendritic GJ	EM	Hamzei-Sichani et al. 2012	Gap junction/ reticular gap junction, previously called "anastomosing" gap junctions
Cx36	Spinal cord	Sexually dimorphic motor nuclei	IF	Bautista et al. 2014	Gap junction
Cx36 (probably, but not studied specifically)	Olfactory bulb	Periglomerular cell dendrites	EM	Kosaka & Kosaka 2005	Gap junction
Cx36 (probably, but not studied specifically)	Olfactory bulb	Dendrites of some interneurons different from periglomerular cells	EM	Kosaka & Kosaka 2005	Gap junction
Cx36	Cerebellar input layer	Golgi cells	Patch clamp, EM	Vervaeke et al. 2010, Szoboszlay	Gap junction/ gap junction plaque

				et al. 2016	
Cx36	Mesencephalon	Trigeminal nucleus	Patch clamp, IF	Curti et al. 2012	Gap junction/ gap junction plaque
Cx36	Auditory system	Anteroventral cochlear nucleus bushy cells	IF	Rubio & Nagy 2015	Gap junction/ gap junction plaque
Cx36	Auditory system	Dorsal cochlear nucleus fusiform cells	IF	Rubio & Nagy 2015	Gap junction/ gap junction plaque
Cx50	Retina	A-type horizontal cells	IF	O'Brien et al. 2006	Gap junction/ gap junction plaque
Cx50	Retina	A-type horizontal cells	EM, IF	Dorgau et al. 2015	Gap junction/ gap junction plaque
Cx36, Cx45	Retina	Inner plexiform layer	EM	Kamasawa et al. 2006, Li et al. 2008	Gap junction/ gap junction plaque

Astrocyte-neuron GJCs

Cx32, Cx26 (astrocyte & neuron)	Brainstem	Locus coeruleus	EM, patch clamp	Alvarez-Maubecin et al. 2000	Gap junction
Cx43 (astrocyte & subpopulation of neurons)	Cerebral cortex	Visual, parietal, frontal cortices	IF, EM	Nadarajah et al. 1996	Gap junction, gap junctional plaques, long junction, reflexive gap junction
Cx43 (satellite glial cells, SGCs) Cx26, Cx30, Cx30.2, Cx36 or Cx45 (neuron)	Peripheral trigeminal ganglia ex vivo	Between SGCs and neurons	Patch clamp, LY dye tracing	Suadicani et al. 2010, Spray et al. 2019	Gap junction
Cx43 (astrocyte) Cx32 (neuron)	Hypothalamus	Supraoptic nucleus	EM, IHC	Duan et al. 2004	Gap junction

Astrocyte-oligodendrocyte (OD) GJCs

Cx43 (astrocyte)/ Cx47 (OD)	Cerebral cortex, (hypo)thalamus	details not available	IF	Nagy et al. 2003	Gap junction, gap junction plaques
Cx43 (astrocyte)/ Cx47 (OD)	Neocortex	Layers III–VI	IF	Wasseff & Scherer 2011	Gap junction
Cx43 (astrocyte)/ Cx47 (OD)	Heterologous expression system	Cell lines (Neuro2A, HeLa)	IF, patch clamp	Orthmann-Murphy et al. 2007	Gap junction, junctional plaques
Cx30 (astrocyte)/ Cx32 (OD)	Heterologous expression system	Cell lines (Neuro2A, HeLa)	IF, patch clamp	Orthmann-Murphy et al. 2007	Gap junction, junctional plaques

Cx43 (astrocyte)/ Cx47 (OD)	Spinal cord white matter	Node of Ranvier	EM, IF	Kamasawa et al. 2005	Conventional crystalline “plaques”, non-crystalline “plaques”, “string” gap junctions, “ribbon” gap junctions, “reticular” gap junctions
Cx43 or Cx30 (astrocyte)/ Cx47 (OD)	Heterologous expression system, primary astrocyte culture	Cell line (HeLa), primary astrocyte	IF	Magnotti et al. 2011	Gap junction, junctional plaques
Cx30 or Cx26 (astrocyte)/ Cx32 (OD)	Heterologous expression system, primary astrocyte culture	Cell line (HeLa), primary astrocyte	IF	Magnotti et al. 2011	Gap junction, junctional plaques

SI References

Alvarez-Maubecin, V., Garcia-Hernandez, F., Williams, J.T. & Van Bockstaele, E.J. Functional coupling between neurons and glia. *J. Neurosci*. **2000**, *20*, 4091-4098;

Bautista, W., Nagy, J.I. Connexin36 in gap junctions forming electrical synapses between motoneurons in sexually dimorphic motor nuclei in spinal cord of rat and mouse. *Eur. J. Neurosci*. **2014**, *39*, 771-787;

Bautista, W., McCrea, D.A., Nagy, J.I. Connexin36 identified at morphologically mixed chemical/electrical synapses on trigeminal motoneurons and at primary afferent terminals on spinal cord neurons in adult mouse and rat. *Neurosci*. **2014**, *263*, 159-180;

Christie, J.M. Cx36 mediates spike synchrony in olfactory bulb glomeruli. *Neuron* **2005**, *46*, 761-772;

Curti, S., Hoge, G., Nagy, J.I., Pereda, A.E. Synergy between electrical coupling and membrane properties promotes strong synchronization of neurons of the mesencephalic trigeminal nucleus. *J Neurosci*. **2012**, *32*, 4341-4359;

Dorgau, B., Herrling, R., Schultz, K., Greb, H., Segelken, J., Ströh, S., Bolte, P., Weiler, R., Dedek, K., Janssen-Bienhold, U. Connexin50 couples axon terminals of mouse horizontal cells by homotypic gap junctions. *J. Comp. Neurol.* **2015**, *523*, 2062-2081;.

Dosztányi, Zs., Fiser A., Simon, I. Stabilization centers in proteins: identification, characterization and predictions. *J. Mol. Biol*. **1997**, *272*, 597-612.

Duan, L., Yuan, H., Su, C.J., Liu, Y.Y., Rao, Z.R. Ultrastructure of junction areas between neurons and astrocytes in rat supraoptic nuclei. *World J. Gastroenterol*. **2004**, *10*, 117-121;

Hamzei-Sichani, F. Davidson KG, Yasumura T, Janssen WG, Wearne SL, Hof PR, Traub RD, Gutiérrez R, Ottersen OP, Rash JE. Mixed electrical-chemical synapses in adult rat hippocampus are primarily glutamatergic and coupled by Connexin-36. *Front. Neuroanat*. **2012**, *6*, 13;

Kamasawa N, Sik A, Morita M, Yasumura T, Davidson KG, Nagy JI, Rash JE. Connexin-47 and connexin-32 in gap junctions of oligodendrocyte somata, myelin sheaths, paranodal loops and Schmidt-Lanterman incisures: implications for ionic homeostasis and potassium siphoning. *Neurosci.* **2005**, *136*, 65-86; DOI: 10.1016/j.neuroscience.2005.08.027.

Kamasawa, N. Furman CS, Davidson KG, Sampson JA, Magnie AR, Gebhardt BR, Kamasawa M, Yasumura T, Zumbrunnen JR, Pickard GE, Nagy JI, Rash JE. Abundance and ultrastructural diversity of neuronal gap junctions in the OFF and ON sublaminae of the inner plexiform layer of rat and mouse retina. *Neurosci.* **2006**, *142*, 1093-1117;

Kosaka, T., Kosaka, K. Intraglomerular dendritic link connected by gap junctions and chemical synapses in the mouse main olfactory bulb: electron microscopic serial section analyses. *Neurosci*. **2005**, *131*, 611-625;

Kosaka, T., Deans, M.R., Paul, D.L., Kosaka, K. Neuronal gap junctions in the mouse main olfactory bulb: morphological analyses on transgenic mice. *Neurosci*. **2005**, *134*, 757-769;

Li, X., Kamasawa, N., Ciolofan, C., Olson, C.O., Lu, S., Davidson, K.G.V., Yasumura, T., Shigemoto, R., Rash, J.E., Nagy, J.I. Connexin45-containing neuronal gap junctions in rodent

retina also contain connexin36 in both apposing hemiplaques, forming bihomotypic gap junctions, with scaffolding contributed by zonula occludens-1. *J. Neurosci*. **2008**, *28*, 9769-9789;

Maeda, S., Nakagawa, S., Suga, M., Yamashita, E., Oshima, A., Fujiyoshi, Y., Tsukihara, T. Structure of the connexin 26 gap junction channel at 3.5 Å resolution. *Nature* **2009**, *458*, 597-602.

Magnotti, L.M., Goodenough, D.A., Paul, D.L. Functional heterotypic interactions between astrocyte and oligodendrocyte connexins. *Glia* **2011**, *59*, 26-34;

Münster-Wandowski, A., Gómez-Lira, G., Gutiérrez, R. Mixed neurotransmission in the hippocampal mossy fibers. *Front. Cell. Neurosci*. **2013**, *7*, 210;

Nadarajah, B., Thomaidou, D., Evans, W.H., Parnavelas, J.G. Gap junctions in the adult cerebral cortex: regional differences in their distribution and cellular expression of connexins. *J. Comp. Neurol*. **1996**, *376*, 326-342;

Nagy, J.I. Evidence for connexin36 localization at hippocampal mossy fiber terminals suggesting mixed chemical/electrical transmission by granule cells. *Brain Res*. **2012**, *1487*, 107-122;

Nagy, J.I., Bautista, W., Blakley, B., Rash, J.E. Morphologically mixed chemical-electrical synapses formed by primary afferents in rodent vestibular nuclei as revealed by immunofluorescence detection of connexin36 and vesicular glutamate transporter-1. *Neurosci.* **2013**, *252*, 468-488;

Nagy, J.I., Ionescu, A.V., Lynn, B.D., Rash, J.E. Connexin29 and connexin32 at oligodendrocyte and astrocyte gap junctions and in myelin of the mouse central nervous system. *J. Comp. Neurol*. 2003, 464, 356-370;

Nagy, J.I., Pereda, A.E., Rash, J.E. On the occurrence and enigmatic functions of mixed (chemical plus electrical) synapses in the mammalian CNS. *Neurosci. Lett.* **2019**, *695*, 53-64;

Nyikos, L., Simon, Á., Barabás, P., Kardos, J. Ligand-specific conformations of an ionotropic glutamate receptor. Protein Engineering. **2002**, *15*, 717-720.

O'Brien, J.J., Li, W., Pan, F., Keung, J., O'Brien, J., Massey, S.C. Coupling between A-type horizontal cells is mediated by connexin 50 gap junctions in the rabbit retina. *J. Neurosci*. **2006**, *26*, 11624-11636;

Orthmann-Murphy, J.L., Freidin, M., Fischer, E., Scherer, S.S., Abrams, C.K. Two distinct heterotypic channels mediate gap junction coupling between astrocyte and oligodendrocyte connexins. *J. Neurosci*. **2007**, *27*, 13949-13957;

Pan, F., Keung, J., Kim, I.B., Snuggs, M.B., Mills, S.L., O'Brien, J., Massey, S.C. Connexin 57 is expressed by the axon terminal network of B-type horizontal cells in the rabbit retina. *J. Comp. Neurol*. **2012**, *520*, 2256-2274;

Rash, J.E. Davidson KG, Kamasawa N, Yasumura T, Kamasawa M, Zhang C, Michaels R, Restrepo D, Ottersen OP, Olson CO, Nagy JI. Ultrastructural localization of connexins (Cx36, Cx43, Cx45), glutamate receptors and aquaporin-4 in rodent olfactory mucosa, olfactory nerve and olfactory bulb. *J. Neurocytol.* **2005**, *34*, 307-341;

Rubio, M.E.; Nagy, J.I. Connexin36 expression in major centers of the auditory system in the CNS of mouse and rat: Evidence for neurons forming purely electrical synapses and morphologically mixed synapses. *Neurosci.* **2015**, *303*, 604-629;

Simon, Á., Dosztányi, Zs., Magyar, Cs., Szirtes, G., Rajnavölgyi, É., Simon, I. Stabilization centers and protein stability. *Theor. Chem. Acc.* **2001**, *106*, 121-127;

Simon, Á., Magyar, Cs., Héja, L., Kardos, J. Peptide binding sites of connexin proteins. *Chemistry* 2, **2020**, 662-673;

Spray, D.C., Iglesias, R., Shraer, N., Suadicani, S.O., Belzer, V., Hanstein, R., Hanani, M. Gap junction mediated signaling between satellite glia and neurons in trigeminal ganglia. *Glia* **2019**, *67*, 791-801;

Suadicani, S.O., Cherkas, P.S., Zuckerman, J., Smith, D.N., Spray, D.C., Hanani, M. Bidirectional calcium signaling between satellite glial cells and neurons in cultured mouse trigeminal ganglia. *Neuron Glia Biol.* **2010**,. *6*, 43-51;

Szoboszlay, M., Lőrincz, A., Lanore, F., Vervaeke, K., Silver, R.A., Nusser, Z. Functional properties of dendritic gap junctions in cerebellar Golgi cells. *Neuron*, **2016**, *90*, 1043-1056;

Vervaeke, K., Lorincz, A., Gleeson, P., Farinella, M., Nusser, Z., Silver, R.A. Rapid desynchronization of an electrically coupled interneuron network with sparse excitatory synaptic input. *Neuron* 2010, 67, 435-451;

Vivar, C., Traub, R.D., Gutiérrez, R. Mixed electrical-chemical transmission between hippocampal mossy fibers and pyramidal cells. *Eur. J. Neurosci*. **2012**, *35*, 76-82;

Wasseff, S.K., Scherer, S.S. Cx32 and Cx47 mediate oligodendrocyte:astrocyte and oligodendrocyte:oligodendrocyte gap junction coupling. *Neurobiol. Dis*. **2011**, *42*, 506-513;
